# An Extraordinary Injury

**Published:** 1888-03

**Authors:** 


### AN EXTRAORDINARY INJURY.

The infliction of fatal injury to the brain by the thrusting of pointed objects beneath or through the upper eyelid, through the orbital plate into the brain, has occurred a number of times. Baby-farmers have been known to procure the death of their charges by pushing needles in, and not long since an irascible "fare" thrust his stick some four inches into the brain of a cab driver in the same way. The effect of such an injury is generally very prompt, and within an hour or two—even if not at once—serious symptoms manifest themselves. A curious exception to this rule was the subject of an inquiry last week at the London Hospital, the victim being a commercial traveler 32 years of age. Until the last few weeks the deceased is stated to have been in good health, and to have kept a set of books most accurately. He was admitted into the hospital complaining of a pain in his head, and feeling drowsy. On the 10th inst., symptoms of apoplexy (?) appeared, and he died a few hours later. On making a post-mortem examination of the brain, an abscess the size of a turkey's egg was discovered at the base, evidently not of recent formation, inside which was a penholder and nib, measuring altogether some three inches in length. This foreign body must have been in its position for some considerable time, it being imbedded in bone. No trace of injury to the corresponding eye or nostril could be detected. The widow of the deceased had never heard him allude to any injury of the kind, and it is quite unknown how and when it was inflicted. The pen and nib were of the ordinary school pattern, and there is nothing to show that the injury was not inflicted years ago when the deceased was at school. Altogether, it is a very remarkable case and demonstrates the extreme tolerance of the brain to a very serious injury, and to the presence of a foreign body under certain circumstances. It is fortunate, in one sense, that the deceased died in a hospital; in private practice his death would have been certified as due to apoplexy, or, in case of an inquest, to "visitation of God."—*Medical Press and Circular.*

